# Development of spontaneous tumours and intestinal lesions in *Fhit* gene knockout mice

**DOI:** 10.1038/sj.bjc.6602182

**Published:** 2004-10-05

**Authors:** T Fujishita, Y Doi, M Sonoshita, H Hiai, M Oshima, K Huebner, C M Croce, M M Taketo

**Affiliations:** 1 Department of Pharmacology, Graduate School of Medicine, Kyoto University, Yoshida-Konoé-cho, Sakyo-ku, Kyoto 606-8501, Japan; 2 Laboratory of Biomedical Genetics, Graduate School of Pharmaceutical Sciences, University of Tokyo, Tokyo 113-0033, Japan; 3 Department of Pathology and Biology of Diseases, Graduate School of Medicine, Kyoto University, Yoshida-Konoé-cho, Sakyo-ku, Kyoto 606-8501, Japan; 4 Kimmel Cancer Institute, Jefferson Medical College, Philadelphia, PA 19107, USA

**Keywords:** fragile histidine triad gene (*FHIT*), tumour suppressor gene, intestinal polyps, intestinal lesions, Apc, *β*-catenin

## Abstract

The fragile histidine triad (*FHIT*) gene is frequently inactivated in various types of tumours. However, the system-wide pathology caused by *FHIT* inactivation has not been examined in detail. Here we demonstrate that *Fhit* gene knockout mice develop tumours in the lymphoid tissue, liver, uterus, testis, forestomach and small intestine, together with structural abnormalities in the small intestinal mucosa. These results suggest that Fhit plays important roles in systemic tumour suppression and in the integrity of mucosal structure of the intestines.

The fragile histidine triad (*FHIT*) gene has been identified as a candidate tumour suppressor gene localized in *FRA3B*, the most sensitive common fragile site, at chromosome 3p14.2 (Ohta *et al*, 1996). Chromosomal deletion of the *FHIT*-containing locus or inactivation of *FHIT* is frequently observed in various types of cancers (for a review, see Huebner and Croce, 2003), consistent with a tumour suppressor function in a variety of organs. The Fhit protein carries a proapoptotic activity through a caspase-dependent pathway in human cancer cells, which may contribute to the tumour suppressor activity (Ji *et al*, 1999; Ishii *et al*, 2001; Roz *et al*, 2002).

Previously, we have demonstrated that *Fhit* mutant mice develop tumours spontaneously in lymphatic tissues, sebaceous glands, liver, stomach, colonic submucosa, uterus, skin, salivary glands, and parathyroid glands (Zanesi *et al*, 2001). Moreover, *Fhit* knockout mice are susceptible to chemical carcinogen-induced tumour formation in the forestomach (Fong *et al*, 2000), which is reversed by adenoviral transduction of the human *FHIT* gene (Dumon *et al*, 2001). These results are consistent with the tumour suppressor function of *FHIT*. To further characterise tissue types affected by inactivation of Fhit, including nontumorous lesions, we have constructed a second mouse strain with a targeted *Fhit* gene, and conducted a thorough pathological analysis. These *Fhit* mutant mice develop tumours in a variety of tissues, including intestinal adenomatous polyps, and show abnormal tissue building of intestinal mucosa, suggesting avenues for further study of Fhit function in normal tissues.

## MATERIALS AND METHODS

All *in vivo* experiments were carried out with ethical committee approval and met the standards required by the UKCCCR guidelines (Workman *et al*, 1998). The mouse *Fhit* gene-targeting vector was constructed to replace exon 5, containing the translation initiation codon, with a pGK-Neo-bpA cassette. Homologous recombination in ES cells (RW4) was confirmed by genomic Southern hybridisation (data not shown). Germline transmitted F1 (+/−) mice were intercrossed to obtain *Fhit* (+/+), (+/−) and (−/−) progeny. Both males and females of each genotype from 12 to 20 months of age were used for thorough pathological examinations. Tissue samples were first examined under a dissection microscope. Then, all tissues were fixed with 4% paraformaldehyde in phosphate-buffered saline (PBS) for 16 h at 4°C, embedded in paraffin wax, sectioned at 4 *μ*m thickness and stained with H&E. For immunostaining, sections were preincubated with 3% BSA/10% goat serum in PBS for 2 h and incubated with anti-*β*-catenin antibody (Sigma Chemical Co., St Louis, MO, USA) at 5000-fold dilution or with c-Kit antibody (Santa Cruz, CA, USA) at 400-fold. Signals were visualised using the Vectastain Elite Kit (Vector Laboratories, Burlingame, CA, USA). For mutation analysis and microsatellite instability (MSI) analysis, DNA samples were extracted from paraffin-embedded sections using DEXPAT (TaKaRa, Japan). Extracted DNA samples were subjected to PCR in 11 contiguous fragments spanning nucleotides 2750–4830 of exon 15 of the *Apc* gene, where germline and somatic mutations were frequently detected. Amplified DNA samples were sequenced directly with the respective primers using the BigDye Terminator Cycle Sequencing Ready Reaction Kit (Applied Biosystems; Rotkreuz, Switzerland) and ABI Prism 377 DNA Sequencing System (Applied Biosystems). For MSI analysis, four primer sets, *D1Mit4*, *D6Mit59*, *D9Mit67* and *D10Mit2* (http://www.informatics.jax.org), were used. DNA samples extracted from polyps were amplified by PCR. After denaturation (100°C, 5 min), PCR products were loaded onto 5% Long Ranger Gel (TaKaRa) in 8 M urea, and electrophoresed at 150 V for 40 min. The dried gel was scanned in a BAS-1800 (Fujifilm, Japan).

## RESULTS AND DISCUSSION

*Fhit* (+/−) and (−/−) mice were viable, fertile and clinically normal up to 12 months of age. However, upon necropsy, tumours and abnormal lesions were found in mice older than 12 months, consistent with observations of another *Fhit* mutant strain (Fong *et al*, 2000). Although several types of tumours developed spontaneously even in the age-matched wild-type littermates, overall incidences of tumours and abnormal lesions were significantly higher in both *Fhit* (+/−) and (−/−) mice than in the wild-type littermates (60, 77 and 30% in *Fhit* (+/−), (−/−) and (+/+) mice, respectively; Table 1

**Table 1:** Tumour and abnormality incidence and tumour spectrum

					**Histological tumour types**		
**Genotype**	***n*^a^**	**Age (months)**	**Incidence of tumours and/or abnormal lesions (% of animals)**	**Intestinal lesions (% of animals)**	**Forestomach papilloma**	**Lymphoid malignancies**	**Other neoplasms**
*Fhit* (+/+)	26	12–20	8 (30%)	0	3 (12%)	3 (12%)	1 hepatocellular carcinoma
						2 lymphomas (1 B-cell lymphoma)	1 periosis hepatis
						1 lymphocyte infiltration	1 endometrial hyperplasia
							
							
*Fhit* (+/–)	90	12–20	54 (60%)^b^	19 (21%)	22 (24%)	23 (26%)	1 hepatocellular carcinoma
				6 small intestinal polyps		18 lymphomas (16 B-cell lymphomas)	1 hepatocellular adenoma
				5 fused villi		5 lymphocyte infiltrations	1 liver hemangioma
				8 swollen crypts			2 endometrial hyperplasia
							1 uterine leiomyoma
							1 lung adenoma
							1 Leydig cell tumour
							1 dermoid cyst
							
*Fhit* (–/–)	26	12–20	20 (77%)^b^	7^c^ (27%)	6 (23%)	11 (42%)	2 hepatocellular carcinomas
				4 small intestinal polyps		9 lymphomas (6 B-cell lymphomas)	2 endometrial hyperplasia
				3 fused villi		2 lymphocyte infiltrations	1 GIST^d^
				1 swollen crypts			

a Number of animals.

b *P*<0.05 compared to wild-type controls by chi-squared test.

c One of these mice contained a small intestinal polyp and a fused villi. The other mice contained only one lesion per mouse.

d Gastrointestinal stromal tumour.

). In both *Fhit* (+/−) and (−/−) mice, lymphoid malignancies including B-cell lymphoma and lymphocyte infiltrations, hemangioma and adenoma of the liver, and uterine leiomyoma were found, which is consistent with the previous report (Zanesi *et al*, 2001). Gastrointestinal stromal tumour (GIST) was described in the other *Fhit* mutant strain. Here, we have confirmed the diagnosis by immunohistochemistry for c-Kit protein (Figure 1A

**Figure 1:**
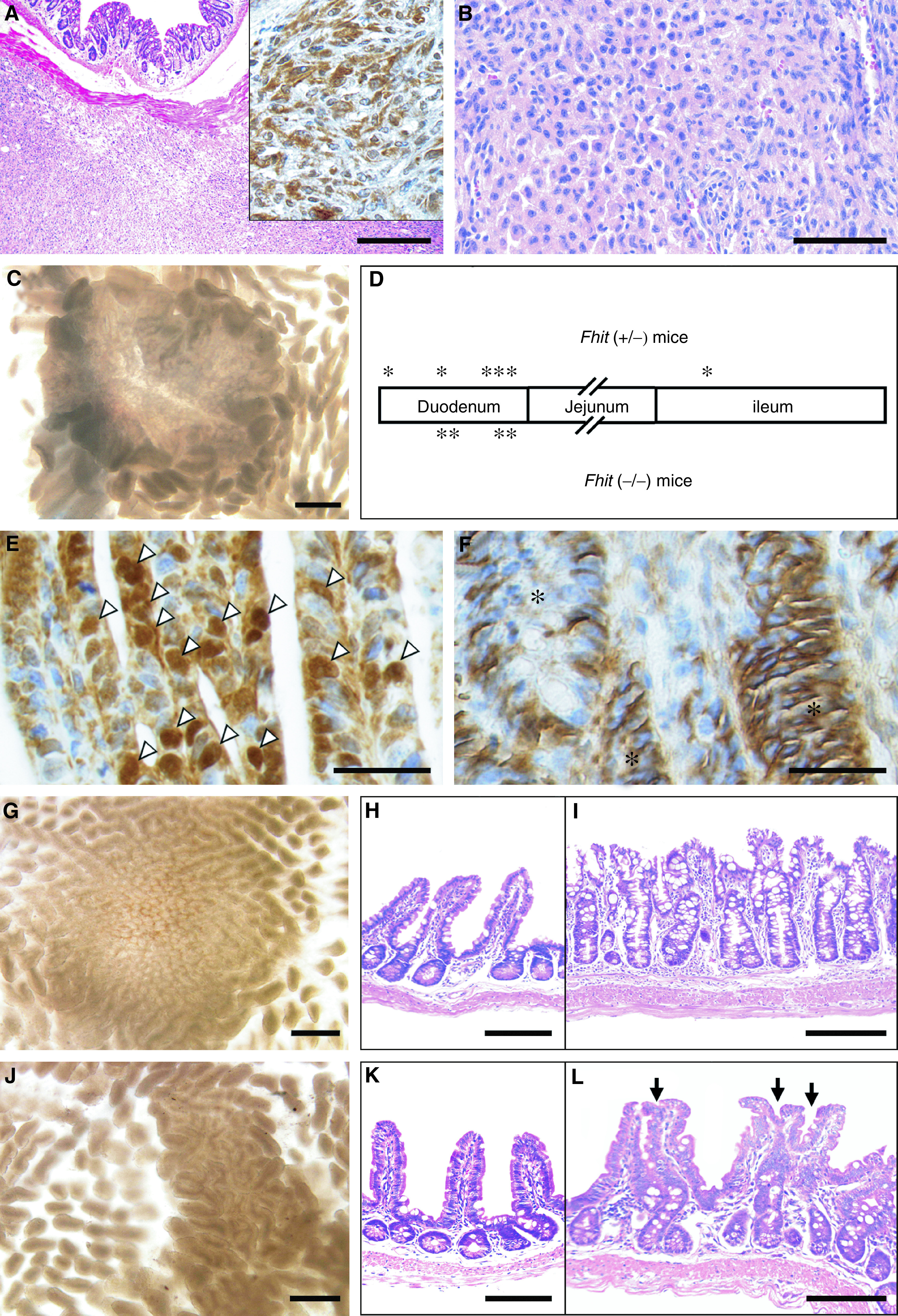
Histology of tumours and abnormal intestinal mucosa in *Fhit*-deficient mice. (**A**) Gastrointestinal stromal tumour in a female *Fhit* (−/−) mouse at 20 months of age. Inset, immunostaining with an anti-c-Kit antibody. (**B**) Leydig cell tumour in a male *Fhit* (+/−) mouse at 15 months. (**C**) Dissection micrograph of a duodenal polyp in a *Fhit* (−/−) mouse at 15 months. (**D**) Schematic distribution of the small intestinal polyps. Asterisks indicate relative locations of the polyps. (**E**, **F**) Immunostaining for *β*-catenin in polyps of a *Fhit* (+/−) mouse at 12 months, and *Fhit* (−/−) at 15 months, respectively. Note the nuclear accumulation of *β*-catenin (white arrowheads) in (**E**) and basolateral staining (asterisks) in (**F**). (**G**, **J**) Dissection micrographs of swollen crypts (ileum) in a *Fhit* (+/−) mouse at 20 months, and fused villi (duodenum) in a *Fhit* (+/−) at 20 months, respectively. (**H**, **K**) Normal ileal mucosa adjacent to the lesions shown in (**I**, **L**), respectively. (**I**, **L**) Histological sections of ileal swollen crypts in a *Fhit* (+/−) mouse at 20 months, and fused villi in a *Fhit* (+/−) at 20 months, respectively. Bars in: (**A**) 500 *μ*m; (**B**) 100 *μ*m; (**C**) 250 *μ*m; (**E**, **F**) 50 *μ*m; (**G**, **J**) 250 *μ*m; (**H**, **I**, **K**, **L**) 200 *μ*m.

inset). In the present study, several additional types of tumours were detected, such as Leydig cell tumour, endometrial hyperplasia and hepatocellular carcinoma (Table 1). However, the last two tumours were observed also in *Fhit* (+/+) mice, and no increase was found in incidence in *Fhit* mutant mice for either tumour type. One Leydig cell tumour was observed in one of 90 *Fhit* (+/−) mice (Figure 1B), although the number is too small to draw a statistical link with Fhit deficiency, and none was found in (−/−) animals.

Interestingly, some adenomatous polyps were found in the small intestine of *Fhit* mutant mice (Figure 1C, D), whereas these polyps have never been observed in wild-type littermates (Table 1). Although the incidence of polyp development in *Fhit* (−/−) was higher (15.4%) than that in *Fhit* (+/−) mice (6.7%), the polyp multiplicity was not different in the two genotypes. These results suggest that tumours develop in *Fhit* (+/−) mice because of *Fhit* haploinsufficiency, either directly or through an indirect mechanism.

Mutations in the gene encoding Apc or *β*-catenin result in intestinal adenomatous polyposis through Wnt signalling activation (Oshima *et al*, 1995; Harada *et al*, 1999). To examine whether the Wnt pathway was activated in the intestinal polyps of *Fhit*-deficient mice, we localised *β*-catenin in nine polyp tissues from both *Fhit* (+/−) and *Fhit* (−/−) mice by immunohistochemistry. *β*-Catenin had accumulated in the nucleus in two polyps of *Fhit* (+/−) mice, indicating Wnt activation in these adenoma cells (Figure 1E). *β*-Catenin was localised to the basolateral side of adenoma cells in the other seven polyps (Figure 1F). By sequence analysis of the *Apc* gene of the two nuclear *β*-catenin-positive polyps, we further found a nonsense mutation in the *Apc* gene at codon 1055 (wt: GAA (Glu) → mutant: TAA (STOP)) in one polyp. This mutation may explain the cause of at least a fraction of the polyps, although other genes may be mutated giving rise to other adenomas. As the intestines were not inspected in detail in the other *Fhit* knockout strain, it is conceivable that similar intestinal lesions existed at low frequencies. These results suggest that an insufficient Fhit level can induce *Apc* gene mutations, which is consistent with the enhanced survival and mutation frequency of Fhit-deficient cells after UVC or mitomycin C damage (Ottey *et al*, 2004). In contrast, MSI was not observed in genomic DNAs from *Fhit* mutant mouse tails or tumours (Fong *et al*, 2000). In the present study, we also examined MSI in cells from two intestinal polyps, using four different sets of markers, and confirmed no MSI in these samples (data not shown). Accordingly, it is possible that inactivation of Fhit results in tumorigenesis through induction of mutations in tumour suppressor genes.

On the other hand, morphological abnormalities in the small intestine, such as swollen crypts (goblet cell hyperplasia, Figure 1G, H) and fused villi (aggregated villi, Figure 1I, J), were observed in both *Fhit* (+/−) and (−/−) mice at similar incidences. Such lesions were not found in the age-matched wild-type mice. Histologically, these lesions consisted of differentiated epithelial cells without any signs of dysplasia. Thus, it is conceivable that Fhit expression is necessary also for the maintenance of normal intestinal architecture, in addition to suppressing tumorigenesis.
